# Different cancers, same target?

**DOI:** 10.18632/aging.101278

**Published:** 2017-08-08

**Authors:** Jaime Villegas, Vincenzo Borgna, Veronica A. Burzio

**Affiliations:** Andes Biotechnologies SpA - Fundación Ciencia & Vida, Santiago, Chile

**Keywords:** ncRNA, mitochondria, antisense

Since its discovery in the early 2000s in mouse and human sperm, the role of long noncoding mitochondrial RNA (lncmtRNAs) remains elusive. It was at the end of this decade that a role in cell proliferation and particularly in tumor cell proliferation was confirmed [1]. Basically, these lncmtRNAs belong to a novel family comprised of two sense (SncmtRNA-1 and -2) and two antisense (ASncmtRNA-1 and -2) members. These transcripts are differentially expressed according to proliferative status; SncmtRNA-1 is expressed in all proliferating cells, while the Antisense transcripts are readily detected in normal proliferating cells but are strongly downregulated in tumor cells. In non-proliferating cells, on the other hand, both Sense and Antisense transcripts are downregulated [1, 2]. This differential expression pattern has been confirmed for a wide array of normal and tumor cells from diverse tissue origins [3]. Interestingly, SncmtRNA-2 has only been detected in oncoviral-immortalized cells, but not in tumor or normal proliferating cells [4]. The few copies of ASncmtRNAs present in tumor cells seem to be essential to tumor cell viability, since knockdown of these transcripts with chemically modified antisense oligonucleotides (ASO) results in massive apoptotic death of tumor cells, preceded by cell cycle arrest. At the molecular level, the effect of the ASO is mediated by strong downregulation of survivin and factors involved in cell migration and invasion [4 – 6]. The same treatment, however, does not affect normal cells, suggesting this approach for the development of an efficient and safe therapeutic strategy for several types of cancer.

We translated our *in vitro* results to the *in vivo* scenario through a series of preclinical assays in mice. Our first results in a syngeneic model of B16-F10 murine melanoma in C57BL6/J mice showed that systemic treatment with 10 doses (every other day) of ASO complementary to the murine ASncmtRNA induced a strong delay in tumor growth [5]. Strikingly, ASO treatment after surgical resection of primary sub-cutaneous tumors completely precluded relapse of the primary tumor and lung and liver metastasis, whereas control ASO-treated mice showed rapid tumor relapse and metastasis [5]. Similar results were obtained in this model with a lentiviral delivery approach of therapeutic sequences [6]. Another syngeneic model we studied was the murine renal adenocarcinoma cell line RenCa in Balb/C mice, in which we observed primarily a delay in tumor growth [7]. This delay was followed, strikingly, by a total remission in subcutaneous tumors, while control mice were sacrificed after reaching the ethical limit of tumor size [7]. Moreover, orthotopic assays of this cell line injected into the subcapsular space of one kidney showed that systemic ASO treatment almost completely prevented tumor growth and drastically diminished lung metastasis, compared to controls [7].

Based on our results, knockdown of the ASncmtRNAs seems to generate a pleiotropic effect on tumor cells, attacking simultaneously several pathways involved in the tumorigenic process, including cell survival, proliferation, invasion and metastasis. Despite this impressive effect, it still remains to be seen if the induction of cell death has a common pathway in different tumor types and if the ASO treatment affects the cancer stem cell population, responsible for relapse. We will also focus in the near future on liquid tumors such as leukemias and lymphomas.

Finally, a Phase I clinical trial, using an ASO targeted to the human ASncmtRNAs, is currently under way (NCT02508441). The trial is designed as an open-label, dose-escalating safety and tolerability study in patients with advanced unresectable solid tumors that are refractory to standard therapy. The result of this trial will be very important in order to continue with a phase Ib and to assess the antitumoral efficacy of this therapy in human cancer patients.
